# Authors’ Reply to: Significant Risks and Responsibilities in the Clinical Use of AI Predictive Models. Comment on: “AI Predictive Model of Mortality and Intensive Care Unit Admission in the COVID-19 Pandemic: Retrospective Population Cohort Study of 12,000 Patients”

**DOI:** 10.2196/82112

**Published:** 2025-09-11

**Authors:** Jose Manuel Ruiz Giardin, Óscar Garnica Alcázar

**Affiliations:** 1 Medicina Interna-Infecciosas Hospital Universitario de Fuenlabrada Fuenlabrada Spain; 2 CIBERINFEC Madrid Spain; 3 Departamento de Arquitectura de Computadores y Automática Facultad de Informática Universidad Complutense de Madrid Madrid Spain

**Keywords:** artificial intelligence, SARS-CoV-2, mortality, predictive model, COVID-19, death, intensive care unit, population study, random forest

We thank the authors of the recent letter [1] for their interest in and thoughtful comments on our article, “AI Predictive Model of Mortality and Intensive Care Unit Admission in the COVID-19 Pandemic: Retrospective Population Cohort Study of 12,000 Patients” [2].

Regarding the low positive predictive value and risk of overtreatment, we acknowledge that our model shows a moderate positive predictive value (25%), but this reflects risk estimation prior to any treatment initiation. Predictions are made at the time of the first clinical evaluation of a patient with SARS-CoV-2 infection. Without subsequent evidence-based interventions—such as oxygen therapy, antivirals, steroids, and interleukin-6 inhibitors—actual mortality might more closely match the model’s predictions. We believe the tool can help optimize guideline-based indications, such as annual vaccination for high-risk patients, early antiviral use regardless of hospitalization status, steroid use in hypoxemia, and tocilizumab for cases with high inflammatory markers. Importantly, the model’s high negative predictive value (98%-99%) makes it valuable for ruling out risk in low-severity patients, aiding resource allocation.

Our model is designed to support—not replace—clinical judgment, and it is best applied for triage, prioritization, and close follow-up, especially in high-demand situations. Any real-world adoption should follow a process of learning and evaluation similar to a clinical trial to assess impact on therapeutic and prognostic outcomes.

We acknowledge the challenge posed by the “black box” in artificial intelligence (AI) models. To address this, we used Shapley additive explanations to provide both global and patient-level interpretability, detailing the most influential variables and interactions (the fifth though ninth figures in the paper [2]). The model outputs a probability of risk, rather than a binary answer, with thresholds adjustable to the clinical context. Clinicians should integrate these probabilities in much the same way as they do other laboratory results.

Regarding the potential impact on insurance access and inequality: this will be particularly important in health care systems where insurers can request and use complete medical records for underwriting; AI use must be accompanied by safeguards to prevent misuse. Although our study took place in Spain’s universal public system—where such risks are minimal—future applications in other contexts require clear ethical frameworks limiting AI outputs to direct patient care. These frameworks should set guidelines for documenting and interpreting AI in clinical practice, supporting physician judgment, and protecting confidentiality. In our study, the model was not applied in real time and outputs were not included in patient records, avoiding therapeutic bias and any influence on insurance or long-term care eligibility. We support strong international regulations to prevent abuse, protect patient rights, and ensure AI does not increase existing inequalities

In summary, we reaffirm that our model is designed as a clinical support tool—not a replacement for physician judgment—and that its validity, interpretability, and careful use have been our top priorities throughout the study. We fully agree that its implementation in any health care system must be accompanied by safeguards, ethical oversight, and clear guidelines to protect patient safety, confidentiality, and equitable access to care.

## Abbreviations

AI: artificial intelligence
